# Tissue microRNA-126 expression level predicts outcome in human osteosarcoma

**DOI:** 10.1186/s13000-015-0329-6

**Published:** 2015-07-21

**Authors:** Wei Liu, Zhong-yuan Zhao, Lei Shi, Wen-dan Yuan

**Affiliations:** College of Basic Medicine, Binzhou Medical University, No. 346 Guanhai Road, Laishan District, Yantai, 264003 Shandong China; Department of orthopedics, Yantaishan Hospital, Yantai, 264000 Shandong China

**Keywords:** Osteosarcoma, microRNA, miR-126, Prognosis

## Abstract

**Background:**

MicroRNA-126 has been found to be consistently under-expressed in osteosarcoma tissues and cell lines compared with normal bone tissues and normal osteoblast cells, respectively. The purpose of the present study was to detect the expression levels of miR-126 in osteosarcoma patients and to further investigate the clinicopathological, and prognostic value of miR-126.

**Methods:**

We recruited 122 patients with osteosarcomas from the Department of Orthopedic Surgery, Yantaishan Hospital between May 2008 and April 2013. The expression level of miR-126 was determined by qRT-PCR. Associations between miR-126 expression and various clinicopathological characteristics were analyzed using the *χ*^2^ test. Survival rate was determined with Kaplan-Meier and statistically analyzed with the log-rank method between groups. Survival data were evaluated through multivariate Cox regression analysis.

**Results:**

miR-126 expression was significantly decreased in osteosarcoma tissues compared to adjacent normal bone tissues (2.421 ± 1.250 vs. 6.212 ± 1.843, *P* = 0.001). We found that low miR-126 expression had significant association with advanced TNM stage (*P* <0.001), distant metastasis (*P* <0.001), and higher tumor grade (*P* = 0.001). Kaplan-Meier survival analysis showed that the miR-126 low-expression group had significantly shorter overall survival time than those with high-expression (log-rank test, *P* = 0.008). Furthermore, multivariate Cox proportional hazards model analysis showed that miR-126 expression was independently associated with overall survival of patients with osteosarcoma (HR = 3.102, 95 % CI: 1.113–9.023, *P* = 0.018).

**Conclusions:**

This is the first study revealing that miR-126 down-expression may be related to the prediction of poor prognosis for osteosarcoma patients, suggesting that miR-126 may serve as a prognostic marker for the optimization of clinical treatments.

## Background

Osteosarcoma is one of the most frequent primary skeletal neoplasms, second only to plasma cell myeloma, in children, adolescents and young adults [1]. Though advances of modern treatments such as surgery, chemotherapy, and the combination of surgery and chemotherapy are improved, long-term survival rate of patients diagnosed with advanced osteosarcoma remains very low [2, 3]. Thus, a better understanding of the biology of this malignancy is necessary to develop novel treatment strategies.

MicroRNAs (miRNAs) represent small, endogenous, non-coding RNA molecules with highly conserved sequences across species in plants, animals, and DNA viruses [4]. miRNAs can regulate gene expression at the posttranscriptional level by binding to the 3′-untranslated regions of their target mRNAs [5]. It has been estimated that miRNAs can regulate as much as 60 % of the human protein coding genes. miRNAs play crucial roles in a number of biological processes, such as embryogenesis, development, cell maintenance, lineage determination, cell proliferation, apoptosis and differentiation [6]. In addition, accumulating studies also show that miRNAs can be dysregulated in various pathological processes, such as human cancers [7]. They function either as oncogenes or as tumor suppressors depending on the role of their target mRNAs [8].

MicroRNA-126 (miR-126) is located on chromosome 9q34.3 within the host gene encoding epidermal growth factor-like domain 7 (EGFL7) [9]. Previously, miR-126 has been found to be consistently under-expressed in osteosarcoma tissues and cell lines compared with normal bone tissues and normal osteoblast cells, respectively [10, 11]. In the present study, we aimed to investigate the clinical significance and prognostic value of miR-126 in osteosarcoma.

## Methods

### Patients and clinical specimens

This study was approved by the Ethical Review Committee of Yantaishan Hospital. All specimens were handled and made anonymous according to the ethical and legal standards and were obtained with patients’ written informed consent. In total, we recruited 122 patients with osteosarcomas from Department of Orthopedic Surgery, Yantaishan Hospital between May 2008 and April 2013. None of the patients enrolled in this study had received any chemotherapy or radiotherapy before surgery. Clinical data was collected from the patients’ database of our institutions. Tumor stage was classified according to the sixth edition of the TNM classification of the International Union against Cancer (UICC). For tissue sample collection, 100 pairs of osteosarcoma tissues and corresponding noncancerous bone tissues were collected from the same patients. The 100 tissue pairs were collected for each patient. The tissues were removed from surgical specimens, immediately transported to the Pathology Laboratory, frozen and stored at −80 °C for RNA extraction. All tissue samples were reviewed by two pathologists, and the clinicopathologic data such as age, sex, site, histological type, tumor grade were summarized in Table 1.

**Table 1:** Association of miR-126 expression with clinicopathological features of osteosarcoma

		miR-126 expression		
Variables	Cases (n)	Low (*n* = 60)	High (*n* = 62)	*P* value
Age (years)				
<18	54	29	25	0.466
≥18	68	31	37	
Gender				
Male	78	37	41	0.707
Female	44	23	21	
Location				
Proximal	72	33	39	0.462
Distal	50	27	23	
TNM stage				
I + II	81	29	52	<0.001
III + IV	41	31	10	
Distant metastasis				
Yes	36	28	8	<0.001
No	86	32	54	
Histological type				
Osteoblastic	55	28	27	0.527
Chondroblastic	32	16	16	
Fibroblastic	27	12	15	
Telangiectatic	8	4	4	
Tumor grade				
Low	55	18	37	0.001
High	67	42	25	

### Quantitative reverse transcriptase PCR (qRT-PCR) assay

The expression of miR-126 in the osteosarcoma and corresponding non-cancer tissues was determined by qRT-PCR assay. Briefly, total RNA was extracted from the tissues using TRIzol reagent (Invitrogen, Carlsbad, CA, USA) according to the manufacturer’s protocol. miRNA expression levels were then quantitated using the TaqMan miRNA real-time RT-PCR kit (Applied Biosystems) according to the manufacturer’s protocol. Data were analyzed using 7500 software v.2.0.1 (Applied Biosystems), with the automatic Ct setting for adapting baseline and threshold for Ct determination. The universal small nuclear RNA U6 (RNU6B) was used as an endogenous control for miRNAs. Each sample was examined in triplicate, and the amounts of PCR products produced were non-neoplasticized to RNU6B. Primers were synthesized by Sangon Biotech (Shanghai, China), and the forward primers for miR-126 and U6 were 5′-tcgtaccgtgagtaataatgcg-3′ and 5′-ctcgcttcggcagcaca-3′, respectively, while the universal qRT-RCR primer included in the One Step PrimeScript® miRNA cDNA Synthesis Kit (Takara, Japan) was used as the reverse primer.

### Statistical analysis

All quantified data were presented as mean ± SD. Differences between groups were calculated using Student’s *t* test. Associations between miR-126 expression and various clinicopathological characteristics were analyzed using the *χ*^2^ test. Survival times were counted from the date of presentation to the date of death or last follow-up time. Survival rate was determined with Kaplan-Meier and statistically analyzed with the log-rank method between groups. Survival data were evaluated through multivariate Cox regression analysis. All *P*-values were determined from 2-sided tests, and statistical significance was based on a *P*-value of 0.05. All statistical analyses were performed using the SPSS 18.0 software (SPSS, Chicago, IL).

## Results

### miR-126 is down-expressed in osteosarcoma tissues

**Fig. 1:**
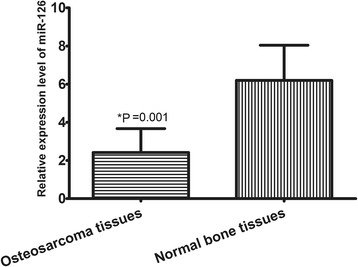
The relative expression of miR-126 in 122 paired human osteosarcoma tissues and their corresponding normal samples

qRT-PCR was used to measure miR-126 expression levels in a total of 122 patients with osteosarcoma. miR-126 expression was significantly decreased in clinical osteosarcoma tissues compared to adjacent normal bone tissues (2.421 ± 1.250 vs. 6.212 ± 1.843, *P* = 0.001, shown in Fig. 1).

### Correlation between miR-126 expression and clinicopathologic features in patients with osteosarcoma

The patients with osteosarcoma were classified into two groups based on the median value (2.06) of relative miR-126 expression. The association between miR-126 expression and clinico-pathologic features in patients with osteosarcoma was analyzed using chi-square test. As shown in Table 1, low miR-126 expression showed significant association with advanced TNM stage (*P* <0.001), distant metastasis (*P* <0.001), and higher tumor grade (*P* = 0.001), but no significant association with patient’s age (*P* = 0.466), gender (*P* = 0.707), location (*P* = 0.462), and histological type (*P* = 0.527). Taken together, these results indicated that down-expressed miR-126 was correlated with the progression and development of osteosarcoma.

### Correlation between miR-126 expression and prognosis in patients with osteosarcoma

**Fig. 2:**
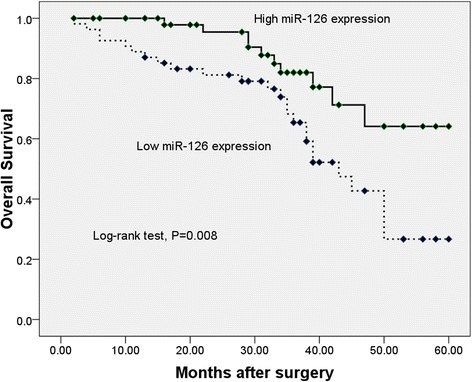
Relationship between miR-126 expression and overall survival time

Kaplan-Meier survival analysis data showed that the miR-126 low-expression group had significantly shorter overall survival time than those with high-expression (log-rank test, *P* = 0.008, shown in Fig. 2). Univariate and multivariate analyses were utilized to evaluate whether the miR-126 expression level and various clinicopathological features were independent prognostic parameters of osteosarcoma patient outcomes. Multivariate Cox proportional hazards model analysis showed that miR-126 expression was independently associated with overall survival of patients with osteosarcoma (HR = 3.102, 95 % CI: 1.113–9.023, *P* = 0.018, shown in Table 2), indicating that miR-126 could be an independent prognostic factor of overall survival for patients with osteosarcoma.

**Table 2:** Univariate and multivariate analyses for prognostic factors

	Univariate analysis			Multivariate analysis		
Variable	Hazard ratio	95 % CI	*P* value	Hazard ratio	95 % CI	*P* value
Sex	0.633	0.462–2.192	0.671	0.781	0.369–1.921	0.762
Age	1.782	0.611–5.829	0.429	1.563	0.673–4.826	0.315
Location	0.881	0.628–5.921	0.317	0.682	0.291–3.267	0.665
Histological type	0.567	0.223–1.982	0.312	0.775	0.478–1.289	0.583
Distant metastasis	3.091	1.982–12.221	0.011	3.558	2.182–12.673	0.005
TNM stage	2.918	1.723–9.273	0.014	3.271	1.923–10.882	0.007
Tumor grade	1.982	1.132–10.923	0.038	2.376	1.234–8.923	0.012
miR-126 expression	2.234	1.012–6.912	0.043	3.102	1.113–9.023	0.018

## Discussion

Osteosarcoma, mainly arising from the metaphysis of the long bones, is the most common primary malignancy of bone in adolescents and young adults, with an estimated worldwide yearly incidence rate of 4 million [12, 13]. Despite current therapeutic strategies combining adjuvant chemotherapy, surgery and sometimes radiotherapy, the prognosis of osteosarcoma patients remains poor, since ~80 % of patients eventually develop recurrent metastatic osteosarcoma following surgical treatment, and the 5-year survival rate of these patients is only 50-60 % [14, 15]. To date, there are no established available biomarkers routinely used to predict the therapeutic outcomes of osteosarcoma reliably. Consequently, exploring more specific prognostic biomarkers to stratify individuals with poor prognosis to benefit from multimodal therapy effectively is an imperative need for advancing the rapeutic strategies for osteosarcoma patients.

Emerging evidence suggests that gene dysregulation, including protein-coding genes, microRNA, and long non-coding RNAs, may affect cellular growth advantage, resulting in progressive and uncontrolled tumor growth and metastasis [16–18]. Effective control of both cell proliferation and cell invasion is critical to the prevention of tumorigenesis and to successful cancer therapy. MiRNAs have been found to be localized to genomic fragilesites and involved in cancer development and progression [19, 20]. Therefore, identification of cancer-associated miRNAs and investigation of their clinical significance and functions may provide a new perspective on the cancer therapy.

MiR-126 is frequently downregulated in a variety of malignancies and acts as a potential tumor suppressor [21–24]. Moreover, low expression of miR-126 has been correlated with poor prognosis in several types of cancer, such as breast cancer, adult T-cell leukemia, and malignant mesothelioma [25–27]. Previously, Xu et al. found that expression level of miR-126 was reduced in osteosarcoma tissues in comparison with the adjacent normal tissues. The enforced expression of miR-126 was able to inhibit cell proliferation in U2OS and MG63 cells, while miR-126 antisense oligonucleotides (antisense miR-126) promoted cell proliferation. At the molecular level, their results further revealed that expression of Sirt1, a member of histone deacetylase, was negatively regulated by miR-126. Their data demonstrated that miR-126 was an important regulator in osteosarcoma [10]. In the study by Yang et al., ectopic expression of miR-126 inhibited cell proliferation, migration and invasion, and induced apoptosis of MG-63 cells. Moreover, bioinformatic prediction suggested that the sex-determining region Y-box 2 (Sox2) was a target gene of miR-126. Using mRNA and protein expression analysis, luciferase assays and rescue assays, they demonstrated that restored expression of Sox2 dampened miR-126-mediated suppression of tumor progression, which suggested the important role of miR-126/Sox2 interaction in tumor progression. Taken together, their data indicated that miR-126 functioned as a tumor suppressor in osteosarcoma, which exerted its activity by suppressing the expression of Sox2 [28]. However, until now, the clinical significance and prognostic value of miR-126 in osteosarcoma have not been investigated. In the present study, qRT-PCR was used to measure miR-126 expression levels in a total of 122 patients with osteosarcoma. We found that miR-126 expression was significantly decreased in clinical osteosarcoma tissues compared to adjacent normal bone tissues. Low miR-126 expression showed significant association with advanced TNM stage, distant metastasis, and higher tumor grade. Kaplan-Meier survival analysis data showed that the miR-126 low-expression group had significantly shorter overall survival time than those with high-expression. Then univariate and multivariate analyses were utilized to evaluate whether the miR-126 expression level and various clinicopathological features were independent prognostic parameters of osteosarcoma patient outcomes. Multivariate Cox proportional hazards model analysis showed that miR-126 expression was independently associated with overall survival of patients with osteosarcoma, indicating that miR-126 could be an independent prognostic factor of overall survival for patients with osteosarcoma.

## Conclusion

In conclusion, this is the first study revealing that miR-126 down-expression may be related to the prediction of poor prognosis for osteosarcoma patients, suggesting that miR-126 may serve as a prognostic marker for the optimization of clinical treatments.
